# Changes in decision-making process for life-sustaining treatment in patients with advanced cancer after the life-sustaining treatment decisions-making act

**DOI:** 10.1186/s12904-021-00759-6

**Published:** 2021-04-27

**Authors:** Hyeyeong Kim, Hyeon-Su Im, Kyong Og Lee, Young Joo Min, Jae-Cheol Jo, Yunsuk Choi, Yoo Jin Lee, Daseul Kang, Changyoung Kim, Su-Jin Koh, Jaekyung Cheon

**Affiliations:** 1 Division of Hematology-Oncology, Department of Internal Medicine, Ulsan University Hospital, University of Ulsan College of Medicine, 877, Bangeojinsunhwando-ro, Dong-gu, 44033 Ulsan, Republic of Korea; 2 Medical Information Center, Ulsan University Hospital, University of Ulsan College of Medicine, Ulsan, Korea; 3 Department of Medical Oncology, CHA Bundang Medical Center, CHA University School of Medicine, 59 Yatap-ro, Bundang-gu, 13496 Seongnam, Republic of Korea

**Keywords:** End‐of‐life process, Life‐sustaining treatment, cancer, Physician orders for life‐sustaining treatment

## Abstract

**Background:**

Cancer is a leading cause of death in Korea. To protect the autonomy and dignity of terminally ill patients, the Life-Sustaining Treatment Decision-Making Act (LST-Act) came into full effect in Korea in February 2018. However, it is unclear whether the LST-Act influences decision- making process for life-sustaining treatment (LST) for terminally ill cancer patients.

**Methods:**

This was a retrospective study conducted with a medical record review of cancer patients who died at Ulsan University Hospital between July 2015 and May 2020. Patients were divided into two groups: those who died in the period before the implementation of the LST-Act (from July 2015 to October 2017, Group 1) and after the implementation of the LST-Act (from February 2018 to May 2020, Group 2). We measured the self-determination rate and the timing of documentation of do-not-resuscitate (DNR) or Physician Orders for Life-Sustaining Treatment (POLST) in both groups.

**Results:**

A total of 1,834 patients were included in the analysis (Group 1, n = 943; Group 2, n = 891). Documentation of DNR or POLST was completed by patients themselves in 1.5 and 63.5 % of patients in Groups 1 and 2, respectively (*p* < 0.001). The mean number of days between documentation of POLST or DNR and death was higher in Group 2 than in Group 1 (21.2 days vs. 14.4 days, *p* = 0.001). The rate of late decision, defined as documentation of DNR or POLST within 7 days prior to death, decreased significantly in Group 2 (56.1 % vs. 47.6 %, *p* < 0.001). In the multivariable analysis, female patients (odds ratio [OR] 0.71, *p* = 0.002) and patients with more than 12 years of education (OR 0.70, *p* = 0.019) were significantly related to a reduced rate of late decision. More than 12 years of education (OR 0.53, *p* = 0.018) and referral to hospice palliative care (OR 0.40, *p* < 0.001) were significantly related to self-determination. Enforcement of LST-Act was related to a reduced rate of surrogate decision-making (OR 0.01, *p* < 0.001) and late decision (OR 0.51, *p* < 0.001). However, physicians with clinical experience of less than 3 years had a higher rate of surrogate decision-making (OR 5.08, *p* = 0.030) and late decision (OR 2.47, *p* = 0.021).

**Conclusions:**

After the implementation of the LST-Act, the rate of self-determination increased and decisions for LST occurred earlier than in the era before the implementation of the LST-Act.

## Background

Despite recent advances in cancer treatment, cancer has become a leading cause of death worldwide. In Korea, it has been the most common cause of death since 1990 and accounted for 27.5 % of all deaths in 2019 [1].

For cancer patients, especially those in the terminal stage, it is important that their personal values and wishes for end-of-life (EOL) care are respected. EOL discussion may reduce aggressive medical care, increase patient and family satisfaction with care, and improve quality of life and survival [2–4]. Advance care planning (ACP) refers to an ongoing process of discussion and review to help patients and families reflect on their goals and values, and to document their preferences for care towards EOL. In the decision-making process for EOL care, patients can recommend the Physician Orders for Life-Sustaining Treatment (POLST) and Advance Directives (AD) to protect autonomy and dignity. Comparatively, patients more frequently participate in decision-making process for LST in Western countries than in Asian countries [5]. Family-centered culture may compel family members to make important decisions about a patient’s treatment, and there are many taboos regarding discussions on death in Asian countries including Korea [6]. As a result, decision-making for life-sustaining treatment (LST) were avoided or postponed, resulting in very late discussions taking place only when the patient’s death was imminent [7, 8]. Therefore, decision-making for LST predominantly occurred between physicians and family members as surrogate decision-makers, excluding patients [9–11].

In 2009, the Supreme Court of Korea adjudicated the withdrawal of a mechanical ventilator on an old female patient in a vegetative state based on her presumed preferences for EOL care. This historic judgment aroused public interest regarding discontinuation of LST and the patient’s right to make decisions about EOL treatments. To advocate for the patients’ wishes and to allow for self-determination in EOL care, the “Life-Sustaining-Treatment Decision-Making Act (LST-Act)” for terminally ill patients came into effect in February 2018 in Korea [12]. Since the implementation of the Act, patients have been able to decide whether they want to apply or suspend LST during the EOL process by documenting POLST during the period when death is imminent with no possibility of recovery, and there is a rapid worsening of symptoms despite treatment. Patients can address preferences for LST by documenting POLST at the terminal stage or during the EOL process, and by documenting AD regardless of their illness.

Before the implementation of the LST-Act, decisions for LST were documented in the do-not-resuscitate (DNR) order, which was not legally effective and may not have entirely reflected the patients’ decisions. As POLST is legal and the LST-Act had penal provisions in case of withholding or withdrawing LST against the patient’s will, physicians became obligated to implement POLST from all patients during the EOL process. However, it is unclear whether the LST-Act influences decision-making process for LST in terms of the rate of self-determination and timing of documentation of DNR or POLST.

Therefore, we conducted this study to investigate the changes in decision-making process for LST in advanced cancer patients during the EOL process after the implementation of the LST-Act.

## Methods

### Patients, study design, and data collection

This was a single-center, retrospective study of cancer patients who died at the Ulsan University Hospital between July 2015 and May 2020.

We assessed patients who had died during two separate periods: the period before the implementation of the LST-Act (from July 2015 to October 2017, “Group 1”) and after the implementation of the LST-Act (from February 2018 to May 2020, “Group 2”). The same inclusion and exclusion criteria were applied to both groups. Cases were limited to patients with cancer at the primary site of head and neck, esophagus, lung, breast, stomach, colorectal, hepatobiliary, and pancreas. We excluded patients with hematologic malignancy, who were younger than 19 years old, or who passed away within 2 weeks of their first visit to Ulsan University Hospital.

We obtained the two groups’ data from the following sources: the clinical data warehouse platform in conjunction with the electronic medical records, Ulsan University Hospital Information of Clinical Ecosystem, the patient characteristics, the type of document for LST (DNR or POLST), decision-makers, date of documentation of DNR or POLST, date of hospice palliative care (HPC) referral, and period between HPC referral and death.

The Institutional Review Board of Ulsan University Hospital approved the study protocol (2020-01-018) but waived the informed consent given the non-requirement of consent in retrospective analyses covered by regulations in Korea. This study was performed in accordance with the ethical standards of the institutional research and the Declaration of Helsinki.

### Definition and outcome measurements

The ‘EOL process’ is when death is imminent with no possibility of recovery, and there is a rapid deterioration of symptoms despite treatment. ‘Terminally ill patients’ are defined as those diagnosed with cancer or other diseases who are expected to die within a few months, and have no possibility of recovery from the underlying disease even with active treatment. ‘LST’ is defined by the LST-Act as cardiopulmonary resuscitation, mechanical ventilation, renal replacement therapy, chemotherapy, and other medical procedures such as antibiotics, transfusions, and artificial nutrition that only extend the duration of the EOL process without any therapeutic effect.

Before the implementation of the LST-Act, the decisions for LST were documented with DNR, and since the implementation of the LST-Act, LST has been decided on using POLST. According to the LST-Act, three steps are required to withhold or withdraw LST in patients during the EOL process. First, two physicians should judge whether the patient is in the EOL process. Second, the patient or patient’s family members should express the patient’s intentions regarding LST by documentation of POLST. If the patient has the ability to make decisions, he/she should express his or her own intentions by in-person documentation of POLST. When the patient is unable to make their own decisions, family members express their intentions for LST on behalf of the patient based on statements made by two or more family members. If it is impossible to verify the patient’s intentions, POLST can be documented through consensus with all adult members of the patient’s family. The final step is clarification of the execution form, describing which LST to withhold or withdraw, as documented by a physician.

To investigate the changes in decision-making process for LST in advanced cancer patients during the EOL process, we measured the changes between the two separate periods as follows: (1) the rates of patients’ self-determination in documentation of DNR or POLST; (2) the number of days since the date of documentation of DNR or POLST; and (3) the rate of documentation of DNR or POLST within 7 days prior to death, reflecting late decision.

### Statistical analysis

We examined between-group associations of demographic and clinical variables using Fisher’s exact test for categorical variables and an independent t-test for continuous variables. Differences in documentation of DNR or POLST and HPC referral between groups 1 and 2 were examined using t-test and chi-square test where appropriate.

The factors affecting surrogate decision-making and late decision (documentation of DNR or POLST within 7 days prior to death) were analyzed using logistic regression analysis. Statistical analysis was performed using the Statistical Package for the Social Sciences (IBM, Armonk, NY, USA) version 22.0. We considered a p-value of less than 0.05 to be statistically significant.

## Results

### Patients’ characteristics

A total of 1,834 patients were included in this analysis: 943 from Group 1 and 891 from Group 2.

There was no significant difference between the two groups in terms of sex, mean age, primary tumor site, years of education, and rates of receiving chemotherapy. The rate of national health insurance coverage was higher in Group 2 with a significant difference between the two groups (95.7 % vs. 93.3 %, *p* = 0.018). In Korea, standard treatments for cancer such as surgery and chemotherapy are reimbursed for citizens; therefore, patients have a little economic burden on cancer treatment regardless of national health insurance. The demographic and clinical characteristics of the patients are summarized in Table 1.

**Table 1:** Baseline characteristics before (Group 1) and after the implementation of the Life-Sustaining Treatment Decisions Act (Group 2)

Characteristics		Group 1 (*n* = 943)	Group 2 (*n* = 891)	*p*-value
		**No. (%)**	**No. (%)**	
**Age at death (Mean ± SD)**		64.9 ± 10.9	66.1 ± 11.1	0.906
**Sex, male**		634 (67.2 %)	564 (63.3 %)	0.078
**Primary site of cancer**	Head and neck cancer	31 (3.3 %)	18 (2.0 %)	0.124
	Esophageal cancer	27 (2.9 %)	15 (1.7 %)	
	Lung cancer	254 (26.98 %)	260 (29.2 %)	
	Breast cancer	48 (5.1 %)	56 (6.3 %)	
	Stomach cancer	106 (11.2 %)	90 (10.1 %)	
	Colorectal cancer	95 (10.1 %)	94 (10.5 %)	
	Hepatobiliary cancer	292 (31.0 %)	253 (28.4 %)	
	Pancreatic cancer	90 (9.5 %)	105 (11.8 %)	
**Education**	≤ 12 years	816 (86.5 %)	781 (87.7 %)	0.935
	> 12 years	122 (12.9 %)	94 (10.5 %)	
**Healthcare insurance**	NHI	880 (93.3 %)	853 (95.7 %)	0.018
	Medical aid	63 (6.7 %)	37 (4.2 %)	
**Received chemotherapy**		475 (50.4 %)	486 (54.5 %)	0.076

*SD* standard deviation; *NHI* National Health Insurance

### Documentation of DNR or POLST and decision‐makers

Table 2 presents the pattern of documentation of DNR or POLST in Groups 1 and 2. A total of 713 patients (75.6 %) in Group 1 and 775 (87.0 %) in Group 2 documented either or both DNR and POLST, respectively (*p* < 0.001).

The rate of self-determination increased significantly in Group 2 (1.5 % vs. 63.5 %, *p* < 0.001). The timing of documentation of POLST or DNR prior to death was prolonged in Group 2 (mean, 14.4 days vs. 21.2 days, *p* = 0.001). The rate of late decision, defined as documentation of DNR or POLST within 7 days of death, decreased significantly in Group 2 (56.1 % vs. 47.6 %, *p* < 0.001).

**Table 2:** Documentation of DNR or POLST before (Group 1) and after (Group 2) the implementation of the Life-Sustaining Treatment Decisions Act

Variables			Group 1 (***n***=943)	Group 2 (***n***=891)	***p-***value
**DNR or POLST documentation**	Completed documentation		713 (75.6%)	775 (87.0%)	<0.001
	Type of document	DNR	713 (100%)	4 (0.5%)	<0.001
		POLST	-	771 (99.5%)	
**Decision makers**^a^	Patients		11 (1.5%)	492 (63.5%)	<0.001
	Surrogate decision-makers		702 (98.5%)	283 (36.5%)	
**DNR or POLST documentation prior to death, day (Mean ± SD)**^a^			14.4 ± 32.5	21.2 ± 43.8	0.001
**DNR or POLST documentation within 7 days of death**^a^			439 (56.1%)	369 (47.6%)	<0.001

^a^ analyzed from cases with documented DNR or POLST (*n*=1488; 783 from group 1, 775 from group 2)

*DNR* do not resuscitate; *POLST* Physician Orders for Life-Sustaining Treatment; *SD* standard deviation

### Referral to hospice palliative care

The rate of referral to HPC was 42.2 and 68.1 % in Groups 1 and 2, respectively (*p* < 0.001). Among the referred patients, 83.9 and 80.2 % provided consent for referral to HPC in Groups 1 and 2, respectively (*p* < 0.001).

The mean duration between HPC referral and death was 36.5 days and 46 days in Groups 1 and 2, respectively (*p* = 0.035) (Table 3).

**Table 3:** Referral to Hospice Palliative Care before (Group 1) and after (Group 2) the implementation of the Life-Sustaining Treatment Decisions Act

Variables	Group 1 (*n* = 943)	Group 2 (*n* = 891)	*p-*value
**HPC referral**	398 (42.2 %)	607 (68.1 %)	< 0.001
**Consent to HPC**^a^	334 (83.9 %)	487 (80.2 %)	< 0.001
**HPC referral to death, day (Mean ± SD)**^a^	36.5 ± 52.9	46.0 ± 79.5	0.035

^a^analyzed from cases referred to HPC (n = 1005; 398 from group 1, 607 from group 2)

*HPC* hospice palliative care; *SD* standard deviation

### The factors associated with late decision and surrogate decision‐making

The factors that were associated with inappropriate decision-making, late decision (documentation of DNR or POLST within 7 days prior to death), and surrogate decision-making are presented in Table 4.

Enforcement of LST-Act was related to a reduced rate of surrogate decision-making (OR 0.01, 95 % confidence interval; CI, 0.01–0.02, *p* < 0.001) and late decision (OR 0.51, 95 % CI, 0.40–0.63, *p* < 0.001). A significant relationship was found between female patients and reduced rate of late decision (odds ratio [OR] 0.71, 95 % CI, 0.57–0.88, *p* = 0.002) as well as between patients with 12 or more years of education and reduced rate of late decision (OR 0.70, 95 % CI, 0.52–0.94, *p* = 0.019). The self-determination rate was higher in patients with 12 or more years of education (OR 0.53, 95 % CI, 0.31–0.90, *p* = 0.018) and those who were referred to HPC (OR 0.40, 95 % CI, 0.29–0.55, *p* < 0.001). Physicians with clinical experience of less than 3 years had a higher rate of decision-making with the surrogate decision-makers than with the patients themselves (OR 5.08, 95 % CI, 1.17–21.99, *p* = 0.030) and late decision (OR 2.47, 95 % CI, 1.14–5.31, *p* = 0.021).

**Table 4:** Factors associated with late decision and surrogate decision-making in those who documented DNR of POLST (*n *= 1488)

Variables	Late decision (documentation of DNR or POLST within 7 days of death)		Surrogate decision-making	
	Odds ratio (95 % CI)	*p*-value	Odds ratio (95 % CI)	*p*-value
**Age (years)**				
≤ 60	1		1	
> 60	1.01 (0.80–1.27)	0.957	1.16 (0.92–1.48)	0.235
**Sex**				
Male	1		1	
Female	0.71 (0.57–0.88)	0.002	0.87 (0.64–1.18)	0.368
**Years of education**				
≤ 12 years	1		1	
> 12 years	0.70 (0.52–0.94)	0.019	0.53 (0.31–0.90)	0.018
**Healthcare insurance**				
NHI	1		1	
Medical aid	0.80 (0.51–1.25)	0.325	0.48 (0.22–1.04)	0.064
**HPC referral**				
No	1		1	
Yes	0.92 (0.76–1.12)	0.397	0.40 (0.29–0.55)	< 0.001
LST-Act				
Before enforcement	1		1	
After enforcement	0.51 (0.40–0.63)	< 0.001	0.01 (0.01–0.02)	< 0.001
**Physician’s years of clinical experience**				
≥ 3 years	1		1	
< 3 years	2.47 (1.14–5.31)	0.021	5.08 (1.17–21.99)	0.030

*CI* confidence interval; *NHI* National Health Insurance; *HPC* hospice palliative care; *DNR* do not resuscitate; *POLST* Physician Orders for Life-Sustaining Treatment; *LST-Act* Life-Sustaining Treatment Decision-Making Act

## Discussion

We found positive changes in decision-making process for LST in advanced cancer patients, such as increased self-determination and earlier decision after the enforcement of the LST-Act.

In Korea, it is still difficult for physicians to talk about death with patients directly because of the reluctance of family members and the family-centered culture in which families make important decisions about a patient’s care [13]. Physicians also have difficulty in delivering bad news directly to patients and experience emotional discomfort when discussing EOL care [14]. Therefore, decisions for LST are frequently delayed, and communication about a patient’s condition occurs between physicians and surrogate decision-makers when it deteriorates [8, 15]. In previous reports in Korea, DNR directives were documented by surrogate decision-makers in almost all cases [9, 11]. However, contrary to the expectations of physicians and family members, patients wanted to hear about their condition directly from the physician [7]. With the aim of increasing patients’ autonomy regarding the right to make their own decisions about LST during the EOL process, the LST-Act came into full effect in February 2018 in Korea. Since the LST-Act’s implementation, the rate of self-determination improved to reach 63.5 % of our study population, which is a significant increase compared to 1.5 % before the implementation of the LST-Act. The self-determination rate in our study is comparable with the rates of previous studies carried out in Western countries, ranging from 23 to 60 % [16–18]. Our findings suggest that the LST-Act might promote patients’ participation in decision-making process for LST.

The timing of decision-making regarding LST occurred earlier than before the LST-Act. The mean time between documentation of DNR or POLST and death increased from 14.4 days to 21.2 days after the implementation of the LST-Act. In a recent retrospective study from Korea, decision-making occurred earlier than before the LST-Act’s implementation, ranging from 17 to 33 days prior to the patient’s death [19]. In Korea, DNR directives were usually documented within a week prior to death, which was too late to reflect patients’ wishes for EOL care [8, 9]. Our results imply that the LST-Act has had a positive effect on earlier decision for LST.

Since the early 2000 s, HPC for terminal cancer patients was introduced and HPC gradually increased in Korea. Provisions for HPC were included in the LST-Act. In our study, the rate of HPC referral increased from 42.2 to 68.1 % over the study period, and the mean time between HPC referral and death increased from 36.5 days to 46.0 days. Findings from previous studies showed that HPC referral was associated with a reduced frequency of aggressive EOL treatment near death and earlier decision for LST [19–22]. In multivariable analysis between inappropriate EOL decision-making (late decision or surrogate decision-making) and patient characteristics, there was a significant decrease in surrogate decision-making in patients who were referred to the HPC team. Referral to HPC is associated with knowledge and awareness for HPC among physicians, patients, and family members [23]. Through HPC referral, patients and family members have improved their understanding of disease status and HPC options. These improvements might lead to earlier decision for LST; thus, patients might have enough time to deliberate about LST and make their own decisions for EOL care. HPC referrals also help physicians feel less of an emotional burden when discussing EOL treatment. Additionally, the rate of self-determination was high at 63.5 % in our study population, compared to the rate of 30 % from other Korean analyses conducted at a similar time [19, 24]. This high rate of self-determination could be interpreted as having been activated by the HPC referral system of Ulsan University Hospital which offers inpatient HPC units and home hospice services. Increased HPC referral might have had positive effects on promoting earlier decision and patient self-determination in our study population.

Multivariable analysis between inappropriate EOL decision-making (late decision or surrogate decision-making) and patient characteristics showed that female patients and patients with more than 12 years of education were less likely to experience late decision. Years of education and HPC referral were related to less surrogate decision-making. Enforcement of the LST-Act was related to a reduced rate of late decision and surrogate decision-making. However, physicians with less than 3 years of professional career experience were related to a higher rate of late decision and surrogate decision-making. In a previous study from Korea, medical oncologists and residents reported that the most common barrier for EOL discussions was prognostication difficulty [25]. Physicians used to learn symptoms and prognosis of terminally ill patients by clinical practices, and less experienced physicians complained of more difficulties in prognosis estimation, compared to senior physicians. As a result, junior physicians tend to initiate EOL discussion when the patient’s condition has deteriorated, and late discussion might be related to surrogate decision-making because patients in the EOL process cannot make a decision. Physicians often receive insufficient training and lack confidence in EOL communication with patients and family members, and they stated that knowledge of the medical, legal, and ethical aspects and communicational preparation were needed in broaching EOL discussion [25, 26]. Previous studies have shown that appropriate education and training improve EOL communication skills and ACP [27]. The LST-Act has increased the documentation of POLST, but physicians are still struggling with discussion for LST. Therefore, physicians need to be trained and supported to discuss EOL care, and programs to integrate EOL conversations and ACP documentation are needed for implementation in routine medical care.

Our study has several limitations. First, it is a study from a single institution, and the decision-making process for LST might be different from those at other medical institutions. Therefore, caution is needed in generalizing the findings. Second, it was a retrospective study, with information sources limited to medical records. Interpretation of data needs to be cautious in causal relationships. Despite these limitations, to our knowledge, this is the first study to compare the decision-making patterns before and after the LST-Act.

## Conclusions

Our study showed that since the implementation of the LST-Act, the self-determination rate rose in clinical practice and decision-making for LST occurred earlier than in the era before the LST-Act. To encourage self-determination and earlier decision, more active interventions, including medical education and training and HPC referral, are needed to ensure that patients’ goals and values are better reflected in the EOL process.

## Data Availability

The datasets used and/or analyzed during the current study are available from the corresponding author upon reasonable request.

## Abbreviations

EOL: End-of-life
ACP: Advance Care Planning
DNR: Do-not-resuscitate
POLST: Physician Orders for Life-Sustaining Treatment
AD: Advance Directives
LST: Life-sustaining treatment
LST-Act: Life-Sustaining Treatment Decision-Making Act
HPC: Hospice palliative care
